# A Validated Methodology for Genetic Identification of Tuna Species (Genus *Thunnus*)

**DOI:** 10.1371/journal.pone.0007606

**Published:** 2009-10-27

**Authors:** Jordi Viñas, Sergi Tudela

**Affiliations:** 1 Laboratori d'Ictiologia Genètica, Departament de Biologia, Universitat de Girona, Girona, Spain; 2 World Wide Fund for Nature, Mediterranean Programme Office, Barcelona, Spain; Erasmus University Medical Center Rotterdam, Netherlands

## Abstract

**Background:**

Tuna species of the genus *Thunnus*, such as the bluefin tunas, are some of the most important and yet most endangered trade fish in the world. Identification of these species in traded forms, however, may be difficult depending on the presentation of the products, which may hamper conservation efforts on trade control. In this paper, we validated a genetic methodology that can fully distinguish between the eight *Thunnus* species from any kind of processed tissue.

**Methodology:**

After testing several genetic markers, a complete discrimination of the eight tuna species was achieved using Forensically Informative Nucleotide Sequencing based primarily on the sequence variability of the hypervariable genetic marker mitochondrial DNA control region (mtDNA CR), followed, in some specific cases, by a second validation by a nuclear marker *rDNA first internal transcribed spacer (ITS1)*. This methodology was able to distinguish all tuna species, including those belonging to the subgenus *Neothunnus* that are very closely related, and in consequence can not be differentiated with other genetic markers of lower variability. This methodology also took into consideration the presence of introgression that has been reported in past studies between *T. thynnus*, *T. orientalis* and *T. alalunga*. Finally, we applied the methodology to cross-check the species identity of 26 processed tuna samples.

**Conclusions:**

Using the combination of two genetic markers, one mitochondrial and another nuclear, allows a full discrimination between all eight tuna species. Unexpectedly, the genetic marker traditionally used for DNA barcoding, *cytochrome oxidase* 1, could not differentiate all species, thus its use as a genetic marker for tuna species identification is questioned.

## Introduction

The genus *Thunnus*, which belongs to the family Scombridae, is comprised of eight species that are commonly known as tunas [1]–[3]. Of these, several species are widely traded at the international level, including the Atlantic bluefin tuna (ABFT; *Thunnus thynnus*), Pacific bluefin tuna (PFBT, *Thunnus orientalis*), Southern bluefin tuna (SBT, *Thunnus maccoyii*), bigeye tuna, (BET, *Thunnus obesus*), yellowfin tuna (YFT, *Thunnus albacares*), and albacore (ALB, *Thunnus alalunga*). Other species of the same family that are also traded as commercial commodities are, among others, skipjack (*Katsuwonus pelamis*) and Atlantic bonito (*Sarda sarda*) [4]. Morphologically, the three bluefin tuna species look very similar, particularly Atlantic and Pacific bluefin tuna, but they are easily distinguishable from bigeye, yellowfin, albacore and skipjack based on external attributes (body shape and other morphometrics, characteristics of the fins, number of gill rakers, etc.). Identification of these species in traded forms, however, which are typically dressed, gilled and gutted, or loin and belly meat, and either fresh/chilled or frozen, is difficult. Especially the three bluefin tuna species, bigeye and yellowfin are almost impossible to distinguish from each other in these forms.

Several protocols have been described for species identification of marine products in recent years, based on different technologies such as isoelectric focusing, high performance liquid chromatography, sodium dodecyl sulphate – polyacrylamide gel electrophoresis, enzyme-linked immunosorbent assay, and starch gel electrophoresis [reviewed in 5,6]. Among these, DNA-based methodologies are one the most promising approaches since they provide very precise tools, and due to their robustness, they can be applied to all the different life stages of marine species. In addition, they can be used on almost all kinds of samples, including whole individuals, fin clips, and canned and dried tissue [5]–[7]. The methodology is usually based on Polymerase Chain Reaction (PCR), which targets a specific genetic marker that is able to discriminate species. Some of the methodologies described to date focus on reducing protocol steps and avoiding DNA sequencing [5]. However, most studies require detailed knowledge of the DNA sequences from target species prior to setting-up the methodology and in insecure cases the final assignation should always be validated afterwards by DNA sequencing [5], [8].

Tuna species can be identified using several genetic markers that have been used in species relationship studies [9]–[14]. However, species misidentification can occur if the genetic marker is not appropriate. For instance, species identification based on nuclear genetic markers cannot distinguish between Atlantic and Pacific bluefin tuna [11]. Furthermore, the low genetic distance among the species belonging to the *Neothunnus* subgenus (*T. albacares*, *T. atlanticus*, *T. tonggol*) [9], [11], [12] can easily confound results if the marker with low genetic variability is used. Therefore, several premises should be considered before attempting the identification of tuna species using mitochondrial genetic markers. Another consideration is that some albacore and Pacific bluefin tuna are so close genetically [9]–[11] that depending on the methodology used (i.e., RFLP-PCR of the mtDNA *Cytochrome oxidase b*) [10], it may be unfeasible to distinguish these two species. Finally, introgression has been described among several tuna species. For instance, about 2–3% of Atlantic bluefin tuna individuals are extremely similar (less than 5% divergence) to Pacific bluefin tuna (NBTAφ) (though in this case the resultant lineages can be separated from proper *T. thynnus* specimens). The same situation occurs vice versa, with about 2–3% of Pacific bluefin tuna individuals having mtDNA extremely similar to Atlantic bluefin tuna (NBTPφ) (about 4.5% genetic distance using mtDNA control region sequencing data) [15]. Introgression also occurs between albacore and Atlantic bluefin tuna, with about 2–3% of Atlantic bluefin tuna individuals having an identical sequence to some albacore. Although several one-step protocols based on mitochondrial DNA that avoid the sequentation of the genetic marker [16]–[25] have been validated for tuna species identification, to our knowledge none of these methodologies can distinguish all the tuna species in a single reaction, and none of them takes into account the possibility of having mtDNA introgression in some of the individuals analyzed.

In this study, we validated a methodology based on Forensically Informative Nucleotide Sequencing (FINS) [26], which takes into account these premises and can fully distinguish among all eight *Thunnus* species. We tested the validity of *Thunnus* species identification for three genetic markers, based on the availability of sequences for all species, previously published phylogenetic studies, and inclusion of nuclear and mitochondrial markers. According to this, we tested mitochondrial DNA control region (mtDNA CR) [9], mtDNA *Cytochrome oxidase subunit I* (*COI*), commonly used in DNA barcoding [12], and the nuclear fragment *rDNA first internal transcribed spacer (ITS1)* [11]. Finally, we applied this methodology to cross-check the species identity of 26 processed tuna samples collected at Japanese markets and restaurants in March and June 2008, as an example of the suitability of the methodology for the routine identification of fish samples. The existence of a reliable genetic methodology to identify species of *Thunnus* at any step of the trade chain is essential for the conservation of some highly overfished species, particularly the Atlantic bluefin tuna which is subject to dramatic levels of illegal fishing [27].

## Results

### Validation of genetic marker for species identification

The comparison of intraspecific genetic variability for each species among molecular markers (Table 1) indicates that the mtDNA CR has about ten-fold greater nucleotide diversity than the other two markers. However, larger sample sizes particularly for the *COI* and nuclear *ITS1* are needed to determine if these differences are meaningful.

**Table 1:** Summary of samples used in the validation methodology and intra and inter-specific levels of genetic variability (*π*, nucleotide diversity).

		Mitochondrial DNA				Nuclear
		*CR*		*COI*		*ITS1*
Species	*n*	*π*	*n*	*π*	*n*	*π*
***T. thynnus***	4	0.023±0.012 (577 [15])	7	0.001±0.001	7	0.004±0.002
**Albacore-like ** ***T. thynnus*** ** (NBTAα)**	5	0.014±0.004 (20 [15])	2	0.002±0.002	2	0.003±0.001
**Pacific-like ** ***T. thynnus*** ** (NBTAφ)**	5	0.006±0.002 (10 [15])	2	0.002±0.002	2	0.002±0.002
***T. orientalis***	3	0.034±0.006 (3, this study)	8	0.007±0.002	4	0.004±0.002
**Atlantic-like ** ***T. orientalis*** ** (NBTPφ)**	2	0.032±0.010 (15)	0	–	0	–
***T. alalunga***	4	0.054±0.027 (134 [28])	6	0.001±0.001	11	0.005±0.002
***T. maccoyii***	3	0.032±0.008 (3 [9])	5	0.003±0.001	4	0.003±0.001
***T. obesus***	4	0.053±0.031 (331 [31])	5	0.002±0.001	12	0.006±0.002
***T. albacares***	5	0.035±0.018 (148 [29])	6	0.001±0.001	13	0.012±0.003
***T. tonggol***	2	(0.059±0.013 (2 [9])	4	0.001±0.001	2	0.003±0.001
***T. atlanticus***	5	(0.037±0.007 (5 [9])	4	0.001±0.001	3	0.003±0.002
**TOTAL**	42	0.125±0.012 (42, this study)	49	0.010±0.002	60	0.028±0.005

For the mitochondrial DNA Control Region (*CR*) and in parenthesis the number of individuals used to estimate the nucleotide diversity and the reference source of the sequences. For the mtDNA *COI* and *ITSI* the nucleotide diversity was estimated using the same data of the phylogenetic analysis.

**Figure 1:**
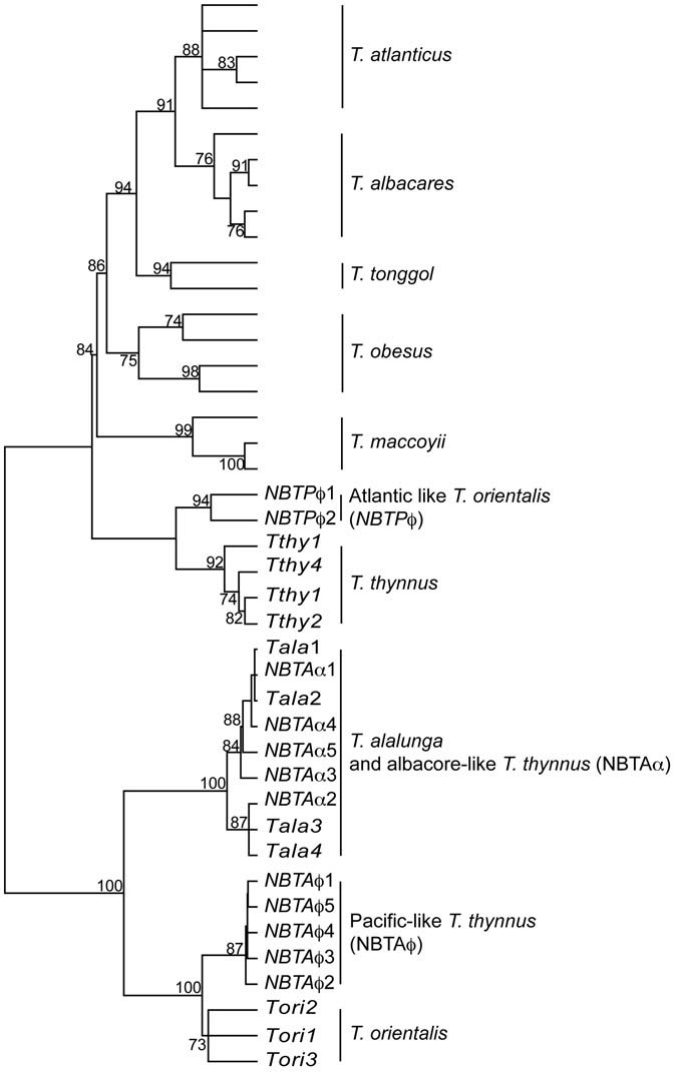
Mitochondrial DNA control region phylogenetic tree. Phylogenetic tree using the 42 mitochondrial control region sequences (mtDNA CR) representing the eight recognized tuna species and including introgressed mtDNA CR sequences of *T. thynnus* and *T. orientalis*. Tree is rooted at midpoint. Numbers above the nodes represent bootstrap support above 70% after 1,000 replicates.

Phylogenetic reconstruction based on the mtDNA CR data resulted in a very consistent phylogenetic tree with monophyletic and well-supported clusters for each species, with bootstrap values ≥ 70% and an average of 5.3 fixed positions (Figure 1). As expected from the study of Alvarado Bremer *et al.* [9], all *Thunnus* species, including the ones belonging to the subgenus *Neothunnus* (*T. albacares*, *T. atlanticus* and *T. tonggol*), were well separated and in consequence easily identifiable by the use of FINS. Furthermore, the mtDNA CR allowed a complete discrimination between the introgressed mtDNA of *T. thynnus* and *T. orientalis* (NBTPφ and NBTAφ) with a bootstrap support of 81% and 92% respectively. However, the mtDNA CR sequences of *T. thynnus* similar to *T. alalunga* (NBTAα) could not be differentiated from *T. alalunga*, as some haplotypes were shared between species.

**Figure 2:**
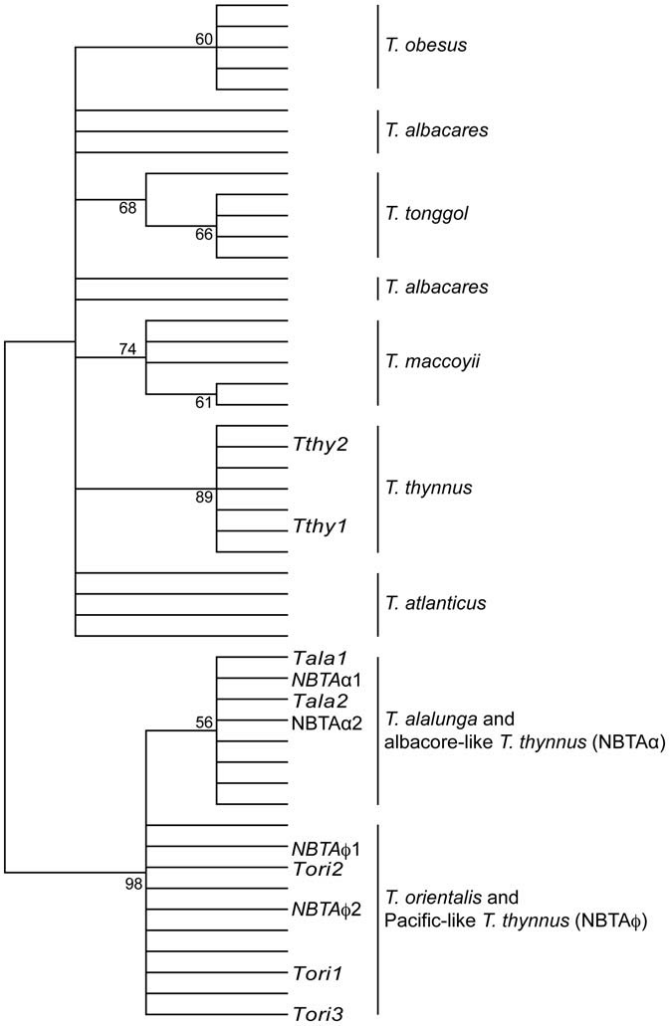
Mitochondrial DNA *cytochrome oxidase 1* (*COI*) phylogenetic tree. Phylogenetic tree using the 49 mitochondrial *COI* representing the eight recognized tuna species and including individuals of *T. thynnus* and *T. orientalis* with introgressed mtDNA. Tree is rooted at midpoint. Numbers above the nodes represent bootstrap support above 60% after 1,000 replicates.

The phylogenetic reconstruction based on *COI* sequences was less consistent than the one based on mtDNA CR (Figure 2). For instance, the three *Neothunnus* species were not grouped together, although all species were clustered separately and in turn, identifiable using this mitochondrial marker. However, *COI* failed to differentiate the *T. thynnus* similar to *T. orientalis* (NBTAφ), with all sequences grouped in a single cluster with a 98% bootstrap support.

**Figure 3:**
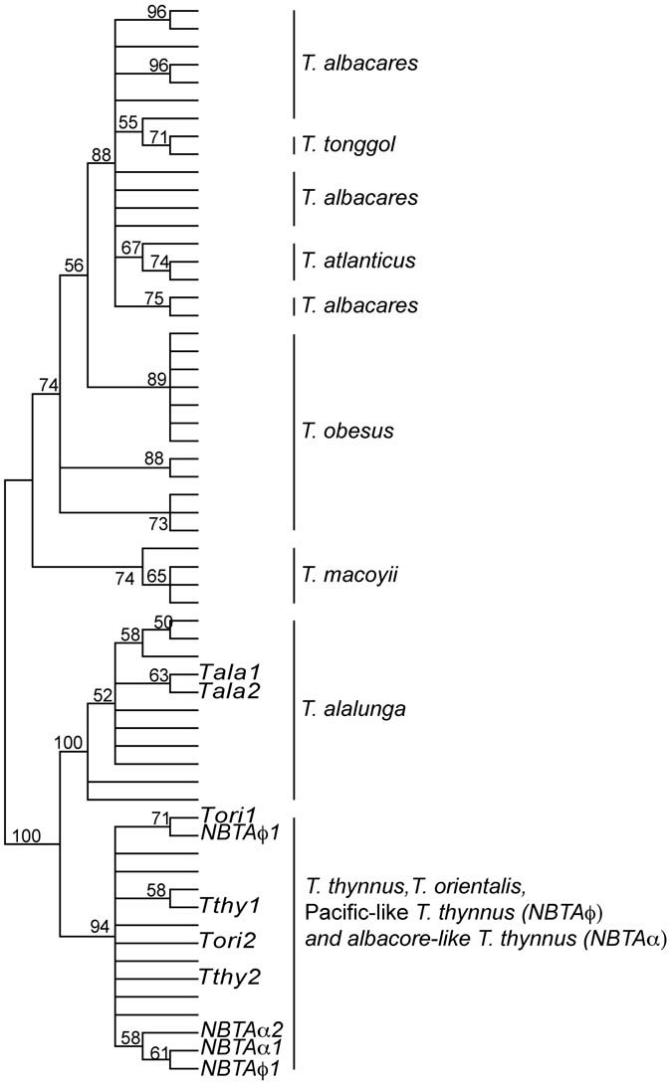
Nuclear *rDNA first internal transcribed spacer* (*ITS1*) phylogenetic tree. Phylogenetic tree using the 60 nuclear *ITS1* sequences representing the eight recognized tuna species and including individuals of *T. thynnus* and *T. orientalis* with introgressed mtDNA. Tree is rooted at midpoint. Numbers above the nodes represent bootstrap support above 50% after 1,000 replicates.

Finally, the gene tree based on the nuclear marker *ITS1* was the least consistent of all genetic trees (Figure 3). This can be seen in the *Neothunnus* species relationship where all three species belonging to this subgenus were grouped in a single cluster and were indistinguishable from each other. A similar situation occurred between *T. orientalis* and *T. thynnus*, as these two species were monophyletic with a bootstrap support of 94%. However, the use of a nuclear marker allowed differentiation between *T. thynnus* with albacore like sequences (NBTAα) from *T. alalunga*. In this case, these introgressed individuals fell in the *T. thynnus + T. orientalis* cluster.

### Species Identification

**Figure 4:**
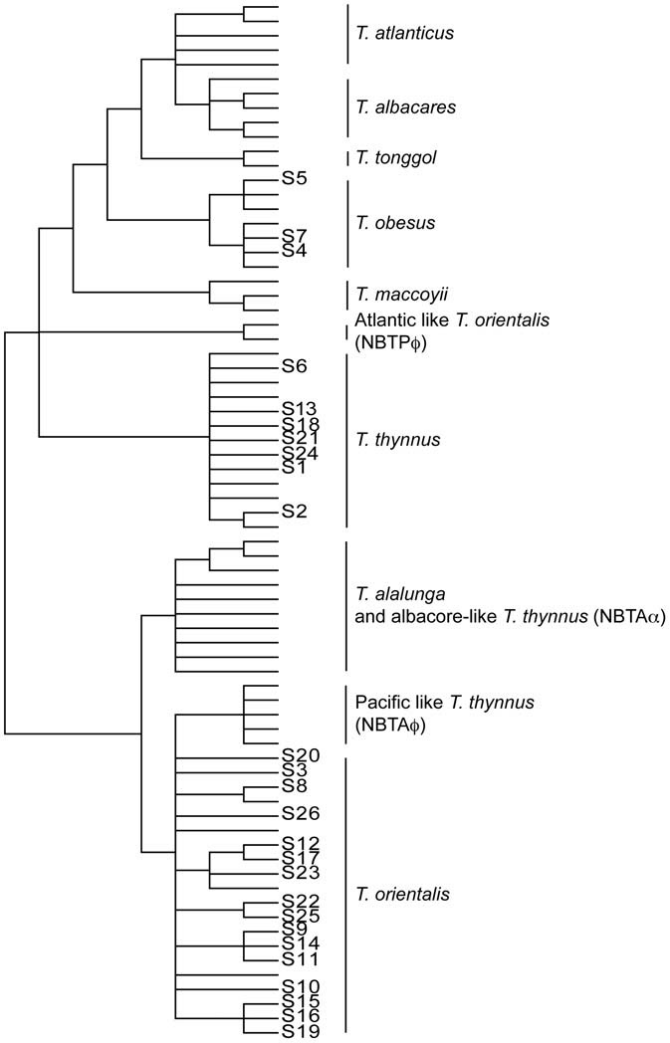
Genetic tuna species identification of unknown samples. Forensically Informative Nucleotide Sequencing of the 26 unknown sampled based on the sequence variability of the mitochondrial control region. Tree is reconstructed based on the information given in Figure 1. Tree is rooted at midpoint.

The 26 tuna samples were evaluated based on mtDNA CR. In all cases the identification was unambiguous (Table 2 and Figure 4) and in consequence, validation with the nuclear *ITS1* was not necessary. Nevertheless, samples S1, S3 and S4 were resequenced with the nuclear *ITS1* (Figure 4). S1 and S3 were clustered in the *T. orientalis* + *T. thynnus* group, and S4 was placed in the *T. obesus* cluster, confirming the identification based on mtDNA CR. Twenty-four of the 26 samples were identified consistently with the species information on the label (Table 2). Two samples, however, S21 and S24, were identified as *T. thynnus*, despite the label on their package showing that they were *T. orientalis*.

**Table 2:** Samples analyzed for species identification with the origin stated in the label and the result after FINS analysis of mitochondrial DNA control region (*mtDNA CR*).

Sample code	Information on Label	*mtDNA CR* species recognition
**S1**	Bluefin (Restaurant Tokyo)	*T. thynnus*
**S2**	Bluefin (Restaurant Tokyo)	*T. thynnus*
**S3**	Bluefin (Restaurant Tokyo)	*T. orientalis*
**S4**	Indian Ocean, Bigeye, Taiwan	*T. obesus*
**S5**	Pacific, Bigeye, Taiwan	*T. obesus*
**S6**	Mediterranean, Bluefin, Spain	*T. thynnus*
**S7**	Pacific, Bigeye, Taiwan	*T. obesus*
**S8**	Japan, farmed ( = *T. orientalis*)	*T. orientalis*
**S9**	Japan, bluefin	*T. orientalis*
**S10**	Japan, bluefin	*T. orientalis*
**S11**	Japan, bluefin	*T. orientalis*
**S12**	Japan, farmed ( = *T. orientalis*)	*T. orientalis*
**S13**	Mediterranean ( = *T. thynnus*)	*T. thynnus*
**S14**	Japan, bluefin	*T. orientalis*
**S15**	Japan, farmed ( = *T. orientalis*)	*T. orientalis*
**S16**	Japan, farmed ( = *T. orientalis*)	*T. orientalis*
**S17**	Pacific, bluefin ( = *T. orientalis*)	*T. orientalis*
**S18**	Mediterranean ( = *T. thynnus*)	*T. thynnus*
**S19**	Japan, farmed ( = *T. orientalis*)	*T. orientalis*
**S20**	Japan, bluefin	*T. orientalis*
**S21**	Japan, farmed ( = *T. orientalis*)	*T. thynnus*
**S22**	Japan, farmed ( = *T. orientalis*)	*T. orientalis*
**S23**	Japan, bluefin	*T. orientalis*
**S24**	Japan, farmed ( = *T. orientalis*)	*T. thynnus*
**S25**	Japan, farmed ( = *T. orientalis*)	*T. orientalis*
**S26**	Japan, bluefin	*T. orientalis*

## Discussion

There is a wide array of molecular methodologies currently available for species identification [6], but they all require that the molecular methodology used is fully validated prior to its application [8]. In the case of the genus *Thunnus* the validation of the genetic marker is even more critical, not only for the importance of these species in the commercial trade, but also due to the observed introgression between *Thunnus* species [9]–[11], [15] that can confound the results, depending on the genetic marker used. In this study, we report on an assessment of the validation of several genetic markers used for *Thunnus* species phylogeny based on the FINS [26] methodology. The selection of genetic markers tested in this study was based on the availability of sequences for these markers for all *Thunnus* species together from previous phylogenetic studies. After an exhaustive literature search, genetic markers were tested: mitochondrial DNA control region (mtDNA CR) [9], mtDNA *Cytochrome oxidase subunit I* (*COI*), commonly used in DNA barcoding [12], and the nuclear fragment *rDNA first internal transcribed spacer (ITS1)* [11]. The mtDNA CR was considered ideal for species validation because previous population-based studies based on this genetic marker had already analyzed hundreds of tuna individuals and had detected mtDNA introgression among *T. thynnus*, *T. orientalis* and *T. alalunga* species [15]. No mtDNA introgression was detected, however, among any other *Thunnus* species (e.g. *T. alalunga* [28]; *T. albacares* [29]; *T. obesus* [30], [31]. Therefore, mtDNA CR has excellent potential as a genetic marker for species identification. Secondly, the mtDNA *COI* marker was tested based on the premise that this marker has been chosen for DNA barcoding and, in principle, has been validated for *Thunnus* species identification [12]. Finally, the use of a nuclear marker was also tested in order to overcome the possible introgression of the mtDNA genome observed between several tuna species. To our knowledge, the *ITS1* was the only nuclear marker used to assess the phylogeny that included all tuna species [11]. Other potential markers, such as the mtDNA *cytochrome oxidase b*, which have been applied for *Thunnus* species identification in phylogenetic studies based on PCR-RFLP [14], sequence data [13], [32], and FINS species identification [14], were discarded due to their lower genetic variability compared to the mtDNA CR [9], and due to the fact that only a limited number of species of the genus *Thunnus* had been characterized. A similar situation occurred with the nuclear gene *Tmo-4C4* [33], with which only three of the eight *Thunnus* species were analyzed.

Genetic marker validation consisted of analyzing individuals with the introgressed signal detected in the study in the study of Alvarado Bremer *et al.* (2005) [15], and comparing them to new sequences of individuals of *T. thynnus*, *T. alalunga* and *T. orientalis* for the three chosen genetic markers. After a close inspection of the phylogenetic tree for the three markers, only the mtDNA CR gene tree allowed a full discrimination of all species (Figure 1), probably as a consequence of its greater genetic variability (Table 1). All species were monophyletic with a strong bootstrap support (≥70%) (Figure 1). Furthermore, the mtDNA CR differentiated the Pacific-like *T. thynnus* (NBTAφ) from the *T. orientalis* (bootstrap support 92%), and the Atlantic-like *T. orientalis* (NBTPφ) from the *T. thynnus* (bootstrap support 81%). On the contrary, neither the mitochondrial *COI* (Figure 2) nor the nuclear *ITS1* (Figure 3) were able to discriminate individuals with introgressed mtDNA from the counterpart species. One question that remains unresolved is the 3% of *T. thynnus* individuals that presents mtDNA CR sequences similar to *T. alalunga* (NBTAα). In this case, any of the two genetic mtDNA markers can distinguish these introgressed *T. thynnus* individuals from *T. alalunga*. However, using the nuclear *ITS1* these individuals are clustered together with *T. alalunga* (Figure 3). Therefore, in the case of a positive identification of individuals as *T. alalunga* using mtDNA CR, a second validation is recommended using the nuclear marker *ITS1* to distinguish between *T. alalunga* or *T. thynnus* with introgressed *T. alalunga* mtDNA.

Another point to consider is the use of *COI* as a genetic maker for *Thunnus* species identification. Hebert *et al.* [34] proposed the use of 648 base pair (bp) portion of the mtDNA *COI* as a standard genetic marker for species identification through the establishment of DNA barcoding. Since then, *COI* has been widely used in fish species identification including *Thunnus* species [12]. However several criticisms have risen mainly related to the use of a single genetic marker [35], [36]. In this study we also raised the question about the appropriateness of the *COI* as the genetic marker for *Thunnus* species identification. As mentioned before, the *COI* is less robust than the mtDNA CR in differentiating all *Thunnus* species. This is more evident in the *Neothunnus* subgenus, where the *T. albacares* sequences were polyphyletic. Furthermore, this genetic marker could not distinguish the introgressed *T. thynnus* sequences similar to *T. orientalis* (NBTAφ), which can be differentiated by the mtDNA CR.

Based on these premises, to prove the applicability of our methodology to commercial samples, we cross-checked the species identity of 26 tuna samples collected at Japanese markets and restaurants, using the mtDNA CR. In all cases the identification was unambiguous (Table 2 and Figure 4), and in consequence the validation of the samples with the nuclear *ITS1* was not necessary. Nevertheless, the individuals S1, S3 and S4 were resequenced with the nuclear *ITS1* and the results coincided with the mtDNA CR. In 92% of the cases, we identified the samples consistently with the information given on the labels. Samples S1 and S2, however, were revealed to be *T. thynnus* despite their labels claiming them to be Japanese farmed tuna (*Thunnus orientalis*). We attributed this difference to mislabeling.

In summary, in this study we propose a method for the complete identification of all eight *Thunnus* species using the FINS approach. This methodology is based in the mtDNA CR sequence variability, followed by a second validation with the *ITS1* nuclear marker only in the cases that the mtDNA CR classifies the individuals as *T. alalunga*. Once these two markers are fully established, an optimization of the protocol to a single-step protocol avoiding the expensive DNA sequencing and based on the detection of SNPs, such as PCR-RFLP, multiplex PCR, Real-time PCR, or other, can be realized. However the high variability of the mtDNA CR (119 parsimony informative sites and an average of approximately 5.3 fixed positions for each cluster of sequences) complicate the set-up of the methodology. Furthermore, we also highlighted the importance of analyzing several individuals, and if possible, representing the full range of the species' distribution, to increase the validity of the method [37]. The methodology described here has an immediate application for the conservation of *Thunnus* species and, particularly, the Atlantic bluefin tuna, *Thunnus thynnus*, which is being subjected to widespread overfishing and illegal trade [27].

## Materials and Methods

### Genetic marker validation

We validated the genetic methodology that is most appropriate for *Thunnus* species identification by testing three different genetic markers: mitochondrial control region (mtDNA CR), mitochondrial *cytochrome oxidase subunit I* gene (*COI*) and the nuclear Internal transcribed spacer region of ribosomal RNA (*ITS1*). All three of these genetic markers have been used in previous studies, to study the phylogenetic relationships of tuna species belonging to the genus *Thunnus* [9], [11], [12]. See Table 1 for a summary of samples used in the validation methodology.

### Source of sequences

The mitochondrial control region data set was comprised of 42 sequences that included the eight recognized species of *Thunnus*. In all possible cases sequences were extracted from population-based studies where hundreds of individuals from all ranges of the species' distribution were analyzed. Only a few representative individuals were included to not collapse the phylogenetic analysis. Thus, the 5 individuals of *T. albacares* were extracted from the population-based study by Ely et al. [29] (Genbank Accession number AY899520–AY899524), the four sequences of *T. obesus* were extracted from the study by Martinez *et al.* [31] (Genbank Accession number DQ126342–DQ126345) and the four *T. alalunga* sequences were from the study by Viñas et al. [28] (Genbank Accession number DQ126342–DQ126345). The 16 *T. thynnus* sequences were extracted from the study by Alvarado Bremer et al. [15]. Of these, six sequences had the real phylogenetic signal of the *T. thynnus* (Genbank Accession number AY650409–AY650414), five were albacore-like *T. thynnus* (from NBTAα1 to NBTAα5) (Genbank Accession number AY650737, AY650494, AY699944, AY650619, AY650594) and five were Pacific-like *T. thynnus* (from NBTAφ1 to NBTAφ5) (Genbank Accession number DQ087593, DQ087541, AY650425, AF390425, AF390384). In the case of *T. orientalis*, two of the *T. orientalis* (*Tori1* and *Tori2*) individuals were newly sequenced since comparison with the sequences of the study by Alvarado Bremer *et al.* [9] gave inconsistent results; the third *T. orientalis* (*Tori3*) (Genbank Accession number AB185022) individual was obtained from the complete mitochondrial sequence of the study by Takashima *et al.* [38]. Furthermore, the *T. orientalis* mtDNA CR data set was complemented with two sequences of the introgressed mtDNA, Atlantic-like *T. orientalis* (NBTPφ1 and NBTPφ2) [15]. Finally, *T. atlanticus* (n = 5), *T. tonggol* (n = 2) and *T. maccoyii* (n = 3) sequences were obtained from the phylogenetic study by Alvarado Bremer *et al*. [9].

For the Cytochrome Oxidase I mitochondrial gene (*COI*) 49 sequences were used that included the eight recognized *Thunnus* species. Sequences of *T. maccoyii* (n = 5) (Genbank Accession number DQ107637–DQ107641), *T. obesus* (n = 5) (Genbank Accession number DQ107629–DQ107630, DQ107642–DQ107644), *T. albacares* (n = 5) (Genbank Accession number DQ107648–DQ107652), *T. tonggol* (n = 5) (Genbank Accession number DQ107632–DQ107636) and *T. atlanticus* (n = 4) (Genbank Accession number DQ107582–DQ107584, DQ107588) were obtained from the study by Ward et al. [12]. For *T. alalunga,* four sequences of the study by Ward et al. [12] (Genbank Accession number DQ107645–DQ107647, DQ107658) were complemented with two newly sequenced individuals (*Tala1* and *Tala2*) (Genbank Accession number GQ414565, GQ414571). These same individuals were also included in the mtDNA CR data set. Similarly, five *T. orientalis* sequences from the study by Ward et al. [12] (Genbank Accession number DQ107590–DQ107592, DQ107631, DQ107581) were complemented with the sequences of *COI* of the same individuals that were also included in the mtDNA CR data set (*Tori1* and *Tori2*) (Genbank Accession number GQ414564–GQ414570), plus the sequence obtained from the complete mitochondrial sequence of the study by Takashima *et al.* [38] (*Tori3*) (Genbank Accession number AB185022). Finally, the *T. thynnus* data set was comprised of four sequences from the study by Ward *et al.* [12] (Genbank Accession number DQ107585–DQ107587, DQ107589), two newly sequenced individuals that were identified as *T. thynnus* (*Thty1* and *Tthy2*) (Genbank Accession number GQ414568, GQ414569) with the mtDNA CR, the sequences of two individuals with mtDNA similar to albacore, albacore-like *T. thynnus* (NBTAα1 and NBTAα2) (Genbank Accession number GQ414567, GQ414572), and two individuals with mtDNA similar to *T. orientalis*, Pacific-like *T. thynnus* (NBTAφ1 and NBTAφ2) (Genbank Accession number GQ414570, GQ414573) [15].

The nuclear segment *ITS1* data set was comprised of 60 sequences. The sequences of *T. maccoyii* (n = 4) (Genbank Accession number AB127399, AB212013–AB212015), *T. obesus* (n = 12) (Genbank Accession number AB127398, AB212016–AB212026), *T. albacares* (n = 13) (Genbank Accession number AB127395, AB212027–AB212038), *T. tonggol* (n = 2) (Genbank Accession number AB127396–AB212039) and *T. atlanticus* (n = 3) (Genbank Accession number AB127397, AB212040–AB212041) were obtained from the study by Chow *et al.* [11]. In addition, the nine *T. alalunga* sequences from the same study (Genbank Accession number AB127402, AB211999–AB212006) were complemented with two new sequences of the same two individuals used for the mtDNA markers (*Tala1* and *Tala2*) (Genbank Accession number GQ414556, GQ414557). In addition, the two *T. orientalis* sequences from the same study (Genbank Accession number AB127400, AB212007) were also complemented with the sequences of the two *T. orientalis* individuals (*Tori1* and *Tori2*) (Genbank Accession number GQ414558, GQ414559). Similarly, the *T. thynnus* data set consisted of five sequences of the study by Chow *et al.* [11] (Genbank Accession number AB127401, AB212009–AB212012) in addition to two new sequences with the “real” *T. thynnus* mtDNA phylogenetic signal already included in both mtDNA markers (*Tthy1* and *Tthy2*) (Genbank Accession number GQ414554, GQ414561), and the sequences of two individuals with the alalunga-like mtDNA phylogenetic signal (NBTAα1 and NBTAα2) (Genbank Accession number GQ414560, GQ414555) and two individuals with the Pacific-like mtDNA (NBTAφ1 and NBTAφ2) (Genbank Accession number GQ414562, GQ414563).

### Species Identification

We collected three samples of tuna from a restaurant (S1 to S3 – Table 2) and 23 from markets (S4 to S26 - Table 2) in Tokyo during March and June 2008. Information on the labels was recorded, and samples were preserved in 96% alcohol until analyzed in the laboratory. Methods for DNA extraction followed the protocol described in Viñas *et al.* [28]. Briefly, total genomic DNA was isolated from each specimen from a small piece of tissue (approximately 100 mg). Tissue was digested overnight at 37°C in a 1.5 ml micro centrifuge tube containing 600 µl of TENS buffer (0.05 M Tris-HCl pH 8; 0.1 M EDTA; 5 M NaCl and 5 M SDS) and 20 µl of Proteinase K (10 mg/ml). Total DNA was extracted with two washes of phenol and one of chloroform isoamyl (24∶1) followed by ethanol precipitation. Finally, the DNA was resuspended with 100 µl of deionized water.

### PCR and sequencing of mtDNA control region, mtDNA cytochrome oxidase 1 (COI) and ITS1 nuclear marker

For the mtDNA CR approximately 450 base pairs (bp) of the first (left) domain of the mitochondrial control region was obtained using the primer combination of L15998 (5′-TAC CCC AAA CTC CCA AAG CTA-3′), with e H-strand primer CSBDH (5′-TgA ATT AGG AAC CAG ATG CCA G-3′) [39]. The amplification was carried out in 25 µl volumes using approximately 50 ng (0.5 µl) of the isolated DNA as a template. In addition, each PCR reaction contained 1X Taq DNA polymerase buffer (supplied by the respective Taq DNA polymerase manufacturer), 1.5–2 mM of MgCl_2_, 200 mM of each dNTP, 10 pMols of each primer and 0.5 U of Taq DNA polymerase (Platinum Taq DNA polymerase, Invitrogen). Thermal cycles involved an initial denaturing step of 5 min at 94°C, followed by 35 cycles of denaturing at 94°C for 45 s, annealing at 50°C for 45 s and extension at 72°C for 1 min. Negative controls were included in all PCR runs to ascertain that no cross-contamination took place. Double-stranded products were checked in agarose gel electrophoresis and purified with the Qiaquick PCR purification kit (Qiagen) and subsequently sequenced with the ABI PRISM BigDye3.1 Terminator Cycle Sequencing Kit (Applied Biosystems) following the manufacturer's recommendations. Finally, sequences were read by an ABI Prism ABI 3130 Genetic Analyzer (Applied Biosystems). Cytochrome oxidase 1 sequences were obtained using the primer combination of universal primers FishF1-5′ TCA ACC AAC CAC AAA GAC ATT GGC AC-3′ and FishR1-5′ TAG ACT TCT GGG TGG CCA AAG AAT CA.-3′ described in Ward *et al.*, (2005) with the same PCR profiles and sequencing procedures as described above. Nuclear *ITS1* sequences were obtained following the protocol described in Chow *et al.* [11] using the primer combination of ITS1-F-5′ TCC GTA GGT GAA ACC TGC GG-3′ with the ITS1-R-5′-CGC TGC GTT CTT CAT CG-3′ using the same reactive described above. PCR profiles consisted of an initial denaturing step of 5 min at 94°C, followed by 35 cycles of touchdown PCR with a denaturing at 95°C for 1 min and initial annealing step of 10 cycles for 1 min at 65°C with a decrease of 1°C/cycle followed by 25 cycles of 55°C and an extension at 72°C for 1 min with a final extension for 10 min at 72°C. The sequencing procedure followed the one described above, but in this case all individuals were sequenced for both strands.

### Sequence analysis

For each genetic marker the sequences obtained were optimized by eye in BIOEDIT [40] in alignment with the orthologous sequences described in the previous section. Intraspecific genetic variability was estimated by nucleotide diversity (π) [41] in MEGA version 4 [42] using the Kimura 2-parameter distance [43]. Phylogenetic relatedness among sequences was reconstructed in MEGA version 4, with neighbor-joining [44] using the Kimura 2-parameter distance. All positions containing alignment gaps and missing data were eliminated only in pairwise sequence comparisons (Pairwise deletion option). Evaluation of statistical confidence in nodes was based on 1,000 non-parametric bootstrap replicates [45].
